# Application of Phase-Reversal Fresnel Zone Plates for High-Resolution Robotic Ultrasonic Non-Destructive Evaluation

**DOI:** 10.3390/s21237792

**Published:** 2021-11-23

**Authors:** Dmitry O. Dolmatov, Daniel Tarrazó-Serrano, German A. Filippov, Antonio Uris, Dmitry A. Sednev

**Affiliations:** 1 School of Non-Destructive Testing, National Research Tomsk Polytechnic University, 7 Savinykh Street, 634028 Tomsk, Russia; ilippovga@tpu.ru; 2 Centro de Tecnologías Físicas, Universitat Politècnica de València, Camí de Vera s/n, 46022 València, Spain; dtarrazo@fis.upv.es (D.T.-S.); Auris@fis.UPV.es (A.U.); 3 National Research Tomsk Polytechnic University, 30 Lenin Avenue, 634050 Tomsk, Russia; sednev@tpu.ru

**Keywords:** ultrasonic nondestructive testing, robotic ultrasonic nondestructive evaluation, ultrasonic imaging, ultrasonic focusing, Phase-Reversal Fresnel Zone Plates, localized coupling

## Abstract

Nowadays the development of automated inspection systems based on six degrees of freedom robotic manipulators is a highly relevant topic in ultrasonic non-destructive testing. One of the issues associated with such development is the problem of acquiring high-resolution results. In this article, the application Phase-Reversal Fresnel Zone Plates is considered for solving this problem. Such acoustic lenses can solve the task of high-resolution results acquisition by using a single unfocused transducer. Furthermore, Phase-Reversal Fresnel Zone Plates can provide the desired focusing depth with the fixed thickness of the coupling layer. It is important in the case of application of devices which provide localized coupling. In this paper a proper design of Phase-Reversal Fresnel Zone Plate was determined according to the conditions of planned experiments. Its efficiency was verified via the Finite Element Method modeling. In all performed experiments the relative error of flaws size estimation did not exceed 6% whereas the signal-to-noise ratio was not lower than 17.1 dB. Thus, experimental results demonstrate that the application of Phase-Reversal Fresnel Zone Plates allowed to obtain results with high lateral resolution and signal-to-noise ratio. These results demonstrate the reasonability of the development of devices that provide localized coupling and use Phase-Reversal Fresnel Zone Plates.

## 1. Introduction

Nowadays, the development and introduction of testing systems based on six degrees of freedom robotic manipulators is the trend in ultrasonic non-destructive evaluation. The application of such manipulators allows the limitations of manual inspections to be overcome. Firstly, six-degrees of freedom robotic manipulators allow for the building of a scanning path with respect to the geometry of the surface of the testing specimen. Such a path implies the manipulation of an ultrasonic probe with six degrees of freedom and provides high repeatability of testing results. Secondly, systems based on six degrees of freedom robotic manipulators are flexible to the changes in objects to control and test conditions. Thirdly, such testing systems are able to perform high-speed scanning of the objects. Finally, the application of such systems allows the testing to be performed in dangerous environments without the necessity of staff presence.

Despite the existing robotic ultrasonic testing systems [1,2,3,4], tool path planning [5,6,7,8,9], data acquisition [10,11], and testing results presentation [12,13] are actual problems in the research and development of such equipment. Furthermore, with the application of robotic testing systems, the task of high-resolution results acquisition can be solved by the application of the focused transducers [14,15] or phased arrays [16,17]. The advantages of phased arrays over the single-element transducers are conditioned by their perfect flexibility and good imaging performance [18]. However, in order to achieve high speed of robotic inspections phased arrays are to be used with expensive multichannel electronic units with high data acquisition rate [19]. Application of single element transducers allows the usage of more simple electronic units which reduce the total price of the robotic systems.

**Figure 1:**
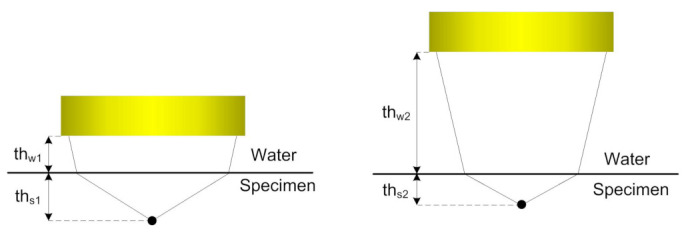
Principle of focused field control in conventional automatic ultrasonic inspections.

Commonly in ultrasonic automated inspections, in order to provide the coupling between the probe and testing object, the latter is placed in an immersion bath. If focused transducers with the concave surface are used, the control of the focusing field can be performed by varying the thickness of the water layer between the probe and testing specimen. The principle is presented in the Figure 1 where $th_{w1}$, $th_{w2}$ are thicknesses of water layer and $th_{s1}$, $th_{s2}$ are obtained depths of focusing inside the specimen obtained by the same transducer application.

However, there are several cases when placing the testing object in an immersion bath is neither possible nor desirable. Such cases include the necessity to test large objects and certain composite materials [20,21]. For these occasions, the devices which provide localized coupling are used including water jet nozzles [2], free-jet nozzles [22] and rubber coupled wheel sensors [23]. Due to the fact that commonly such a device has fixed construction, it is challenging to apply the conventional approach of control of the focused field. In this regard, there is a need to introduce alternative approaches to control the focused field. In general, such approaches should meet several requirements. Firstly, it should provide the desired focusing depth without the necessity to change the thickness of the coupling medium. Secondly, results obtained by this approach application should have high resolution and signal-to-noise ratio. Thirdly, the issues related to manufacturability, cost, size, and weight are important.

According to the pointed requirements, Phase-Reversal Fresnel Zone Plates (PR-FZP) are of special interest. PR-FZP are flat lenses consisting of several concentric rings. Each ring corresponds to Fresnel zone and there is a π phase difference between two consecutive zones. Such arrangement of the lens provides the phase change that generates a constructive interference at the focal distance [24]. Furthermore, PR-FZP good manufacturability is conditioned by the option of their 3D printing [24]. All of this allows to consider PR-FZP as the flexible focusing approach in robotic ultrasonic testing applications.

Thus, the aim of this research is to study the efficiency of PR-FZP for robotic ultrasonic testing applications. The necessity of such study is related to the fact that despite the existed research related to acoustic PR-FZP [24,25], there is insufficient volume of studies related to PR-FZP utilization in pulse-echo ultrasonic nondestructive testing applications. For instance, the efficiency of the PR-FZP when working with reflected ultrasonic signals (echo signals) has not been considered in existing research. Furthermore, one of the basic features of pulse-echo testing is the application of pulsed ultrasonic signals. In this regard, it is necessary to verify the efficiency of PR-FZP when pulsed ultrasonic signals are applied.

In order to achieve the goal of the research, several tasks are to be completed. Firstly, it is necessary to determine a proper PR-FZP design according to the parameters of planned ultrasonic inspection. Secondly, the efficiency of PR-FZP is to be verified via finite element modeling in COMSOL software. Thirdly, PR-FZP with the proper design should be manufactured using 3D printing. Finally, efficiency of manufactured PR-FZP should be checked via in-situ experiments.

## 2. Theory and Determination of Design Parameters of the Lens

As it was mentioned earlier, the design of PR-FZP includes several zones with different acoustic impedance for ultrasonic waves. The main feature of PR-FZP which its design provides phase compensation in zones that contribute destructively to focusing. This can be achieved by the appropriate selection of lens material and its thickness. The material of PR-FZP should provide good transmittance of the ultrasonic waves to the host media. Also, the thickness of PR-FZP is to be selected in order to provide necessary phase correction.

The main parameters of PR-FZP which determine its construction are focal length ($F_{L}$), the central frequency of ultrasonic transducer ($f_{0}$), and the number of Fresnel zones (*N*) which is defined by the size of the applied ultrasonic transducer. Due to the fact that ultrasonic testing through the coupling medium is considered in this study, there needs to be taken into account the propagation of ultrasonic waves through the two media: coupling medium and testing object. In this regard, the focal distance can be evaluated using the following equation:

(1) $$F_{L}=d+F\frac{c_{1}}{c_{2}}$$

where *d* is the thickness of layer of coupling media; $c_{1}$ is the velocity of longitudinal ultrasonic waves in material of controlled object; $c_{2}$ is the velocity of longitudinal ultrasonic waves in material of coupling media; *F* is the desired depth of the focusing in controlled object. For the plane wave incidence, the following equation can be used for the determination of PR-FZP radii [26]:

(2) $$r_{n}=\sqrt{n\lambda F_{L}+{\left(\frac{n\lambda }{2}\right)}^{2}}\;\;n=1,2,3,...,N$$

In order to provide the phase difference equal to a multiple and odd of π between phase-reversal and transparent regions, the appropriate thickness of PR-FZP should be determined using the following equation [24]:

(3) $$t_{h}=\frac{q}{2}\frac{\lambda_{1}\lambda_{2}}{|\lambda_{1}-\lambda_{2}|}$$

where *q* is the design parameter that determines the thickness of all Fresnel regions (q=1,3,5,...), $\lambda_{1}$ is the wavelength in PR-FZP material, and $\lambda_{2}$ is wavelength in host (coupling) medium. Based on Equations (1)–(3), it is possible to determine PR-FZP design parameters based on conditions of planned ultrasonic inspection. These parameters are presented in Table 1.

**Figure 2:**
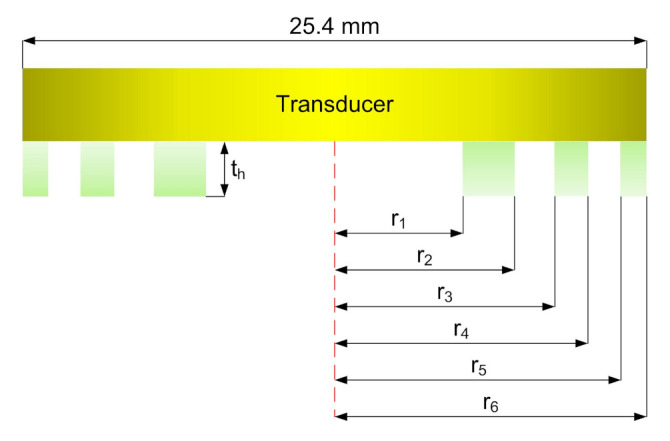
Building scheme of Phase-Reversal Fresnel Zone Plate.

Thus, the Fresnel radii obtained via the Equation (2) are 5.87, 7.33, 8.98, 10.37, 11.60, 12.71 mm for $r_{1}$, $r_{2}$, $r_{3}$, $r_{4}$, $r_{5}$, $r_{6}$, respectively and the most appropriate thickness of PR-FZP obtained through the calculations is 2.19 mm. The Figure 2 presents the building scheme of PR-FZP.

### Lens Efficiency Verification through Numerical Model

In order to verify the performance of the current PR-FZP design, Finite Element Method (FEM) modeling was applied. The acoustic module in commercial software COMSOL was used. Due to the rotational symmetry of the applied mode axisymmetric model has been defined. In system of localized coupling it is common to use the coupling materials with properties close to the water [23,27]. In this regard, in FEM a coupling material with properties close to the water can be considered. Totally three materials should be considered in the model: Polylactic Acid (PLA, the material is planned for usage for PR-FZP manufacturing), material of coupling media, and steel (material of testing object). The sound speeds of 1500, 5900, and 2200 m/s were chosen for coupling media, steel, and PLA, respectively. The density values for the materials was 1000 kg/m${}^{3}$ (coupling material), 7850 kg/m${}^{3}$ (steel) and 1240 kg/m${}^{3}$ (PLA). In the framework of performed simulations transducer surface has been considered as a pressure boundary contour condition. In order to replicate the real performance of the materials for the steel and PLA materials, the Solid-Mechanics module was used. Furthermore, for that materials, linear elastic material contour condition has been applied. To obtain a coherent solution, the multi-physics module was used, so that the contours are perfectly coupled. The selected mesh geometry was triangular. In order to avoid numerical dispersion, the minimum and maximum element size was chosen as $\lambda_{w}$/16 and $\lambda_{w}$/8, respectively ($\lambda_{w}$ is the wavelength in coupling media).

**Figure 3:**
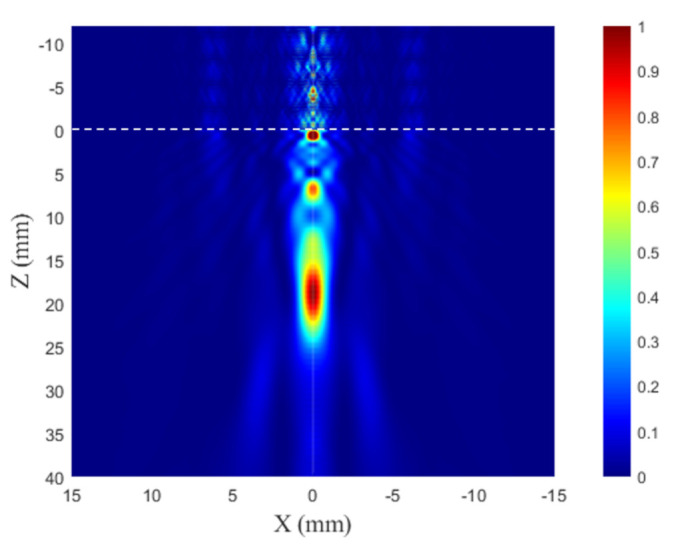
Numerical results for normalized acoustic intensity.

FEM results are presented in Figure 3. The obtained result can be numerically evaluated by using Full Width Half Maximum (FWHM) and Full Length Half Maximum (FLHM) values. The obtained values for FLHM and FWHM were 1.375 mm and 11.127 mm, respectively. FWHM directly refers to lateral resolution. The obtained result for it (1.475 mm) demonstrates that the application of PR-FZP has the ability to obtain the results with high resolution.

## 3. Experimental Set-Up

**Figure 4:**
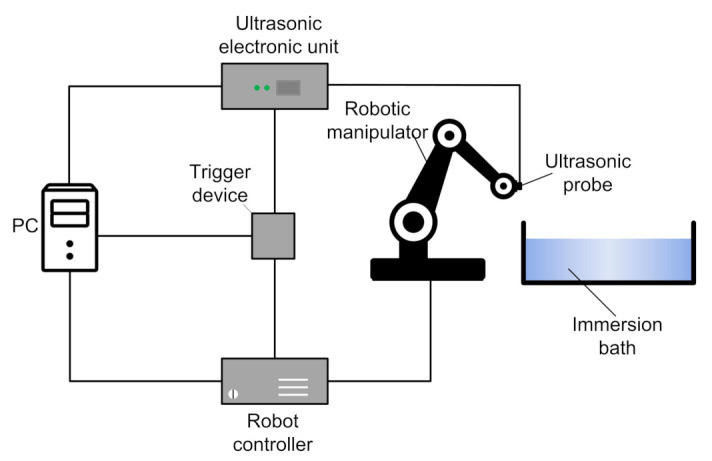
The scheme of experimental set-up.

The verification of the PR-FZP design was performed on the experimental setup which structural scheme is presented in Figure 4.

**Figure 5:**
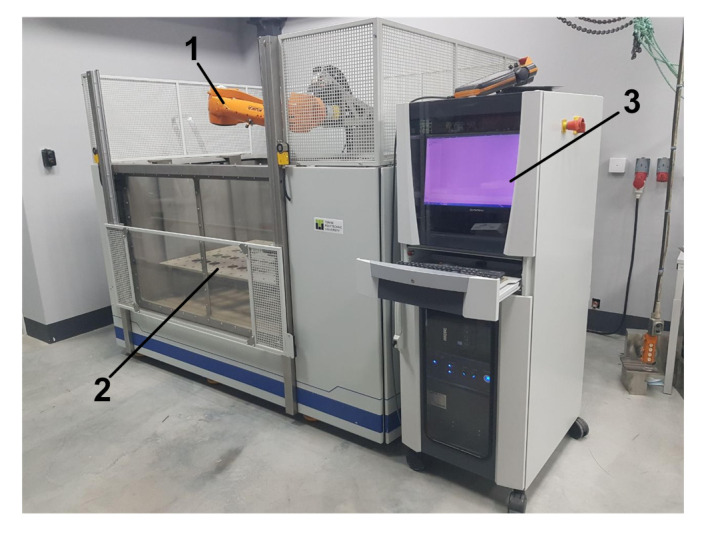
Experimental setup (1—robotic manipulator; 2—immersion bath; 3—control cabinet).

The ultrasonic electronic unit generates the electronic signal for ultrasonic waves excitation by the probe. Also, it receipts, and digitalizes ultrasonic waves and provide their transmission to PC for subsequent processing. In the current experimental set-up, KUKA KR 10 1100 SIX is used as a robotic manipulator. It provides the accurate positioning of the ultrasonic probe at each point of the scanning trajectory. The robot controller provides the control of the robotic manipulator movement according to the selected measurement path. The trigger device is used to initiate the ultrasonic data sampling in the desired point of scanning trajectory via the generation of the strobe impulse to the ultrasonic electronic unit. PC, robot controller, ultrasonic electronic unit, and trigger device are mounted in the control cabinet. The photo of the experimental setup is presented in Figure 5.

**Figure 6:**
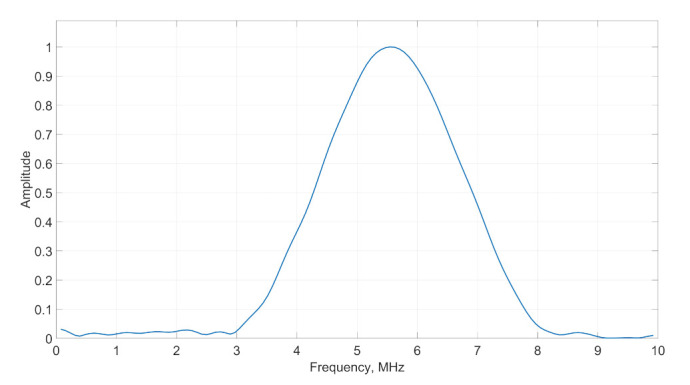
The magnitude of transducer pulse spectrum.

The OLYMPUS A307S-SU transducer was used in experiments. The nominal central frequency of the probe was 5 MHz with the diameter of piezolement is 25.4 mm. The magnitude of the pulse spectrum of the applied transducer is demonstrated in Figure 6. According to pulse spectrum the peak frequency of the transducer was 5.55 MHz and −6 dB bandwidth was 49.3%.

**Figure 7:**
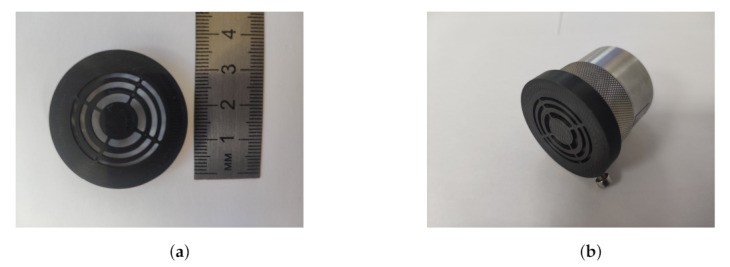
Photographs of the designed and implemented lens. (**a**) Manufactured PR-FZP; (**b**) PR-FZP placement on the probe.

The PR-FZP with determined parameters was manufactured using a 3D printer and PLA filament. Figure 7a demonstrates the manufactured acoustic lens whereas the placement of PR-FZP on the probe is shown in Figure 7b.

**Figure 8:**
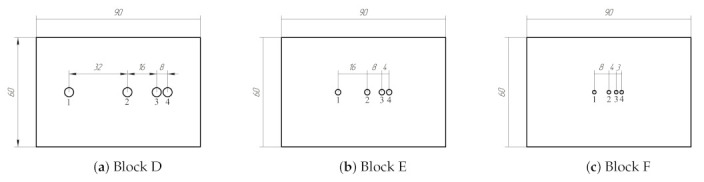
Location of the defects in steel blocks.

In order to evaluate the efficiency of PR-FZP, the set of testing blocks with the thickness of 30 mm containing flat bottom holes was used. The drilling depth of all holes in the specimens was 10 mm. Three testing blocks contain one flaw with varying diameters: 5 mm (block A), 3 mm (block B), and 2 mm (block C). Three testing blocks contain closely spaced flat bottom holes with diameters 5 mm (block D), 3 mm (block E), and 2 mm (block F). The location of flat bottom holes in testing blocks D, E, and F is presented in Figure 8.

Each testing block was scanned with the application of PR-FZP and without it. In all cases, each testing block was scanned with the step of 0.25 mm along both scanning axes. The ultrasonic signals were generated using 200 V negative rectangular electronic signal with the duration of 500 nanoseconds.

## 4. Results and Discussion

**Figure 9:**
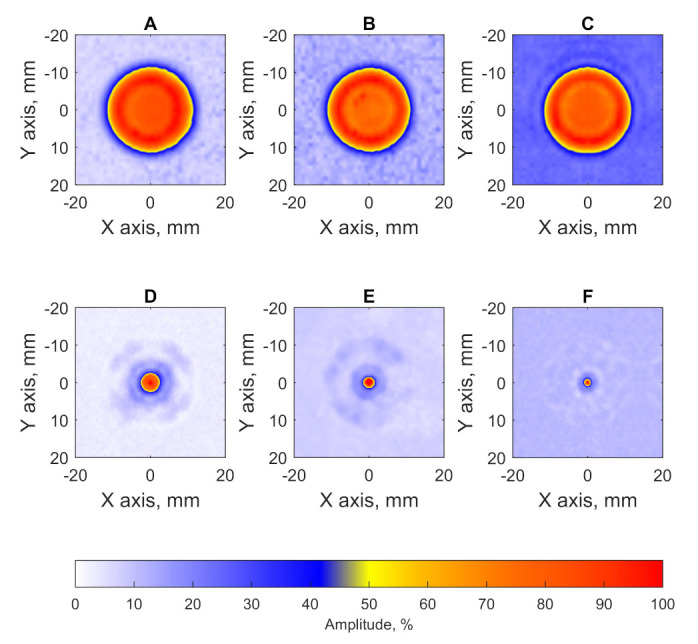
C-scans of samples with single flat bottom holes: (**A**) C-scan of sample A without PR-FZP application, (**B**) C-scan of sample B without PR-FZP application; (**C**) C-scan of sample C without PR-FZP application; (**D**) C-scan of sample A with PR-FZP application; (**E**) C-scan of sample B with PR-FZP application; (**F**) C-scan of sample C with PR-FZP application.

**Figure 10:**
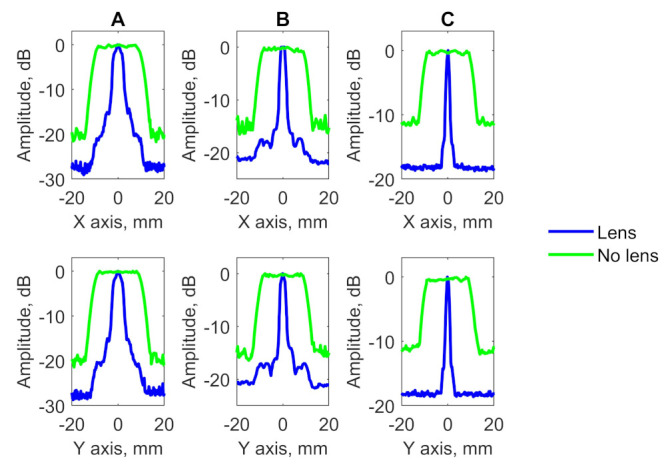
Lateral profiles of results obtained for block (**A**) A, (**B**) B, and (**C**) C on the X and Y axis.

Figure 9 shows the inspection results of testing blocks with a single defect in the form of C-scans. The profiles of obtained imagery are demonstrated in Figure 10. Furthermore, the obtained results can be used for estimating of flaws size via the −6 dB drop method and signal to noise (SNR) using the following equation:

(4) $$SNR=20\cdot \log_{10}\left(\frac{I_{s}}{I_{n}}\right)$$

where $I_{s}$ is a maximum amplitude of signal reflected from the flaw and $I_{n}$ is a maximum amplitude in a region which is away from scatterers [28]. The results of flaws diameter and SNR evaluation are presented in Table 2.

The results demonstrate the efficiency of the PR-FZP application. Application of the developed acoustic lens allowed to obtain the results with improved lateral resolution and signal-to-noise ratio. High lateral resolution allowed to determine the diameters of FBH in testing blocks with high precision.

**Figure 11:**
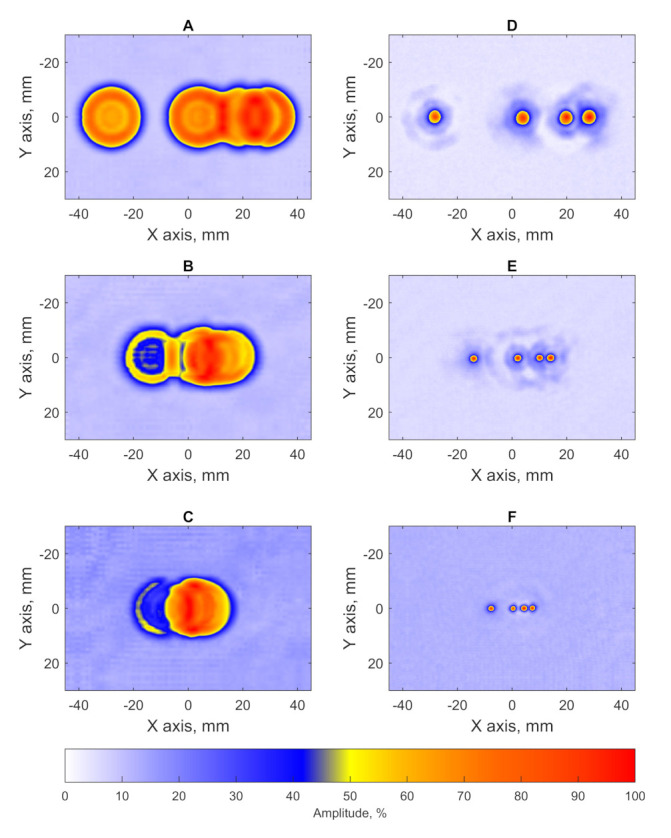
C-scans of samples with close-spaced defect: (**A**) C-scan of sample D without PR-FZP application, (**B**) C-scan of sample E without PR-FZP application; (**C**) C-scan of sample F without PR-FZP application; (**D**) C-scan of sample D with PR-FZP application; (**E**) C-scan of sample E with PR-FZP application; (**F**) C-scan of sample F with PR-FZP application.

**Figure 12:**
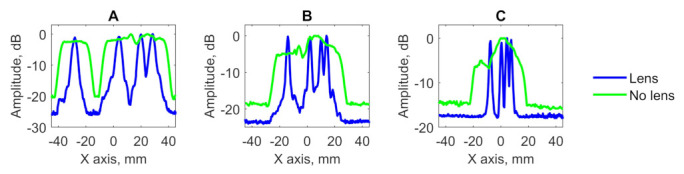
Lateral profiles of results obtained for block (**A**) D, (**B**) E, and (**C**) F on the X axis.

The testing result of blocks D, E and F in the form of C-scans is shown in Figure 11. The lateral profiles of obtained imagery are shown in Figure 12. The results of flaws diameter and SNR evaluation when PR-FZP is applied are presented in Table 3. The designation of defects in Table 3 is given in accordance with Figure 8.

According to the obtained results for the blocks D, E, and F, the defect characterization task can be solved effectively by the application of PR-FZP. According to the obtained results, it is possible to conclude that all closely spaced defects in testing specimens have been resolved. This conclusion can be reached using the obtained lateral profiles of results (Figure 12) at which peaks related to the flaws decrease by at least −6 dB with respect to the peak maximum with lower amplitude [29]. Furthermore, the application of PR-FZP made it possible to obtain C-scans of testing blocks with high resolution and SNR which allowed to estimate the sizes of the flaws with high precision. The results of flaws size and SNR estimation for blocks with closely-spaced defects correspond well with the similar results obtained for the blocks with a single flaw.

## 5. Conclusions

In this paper, the application of PR-FZP for imaging in ultrasonic nondestructive testing has been considered. This interest is associated with their application in robotic ultrasonic testing in conjunction with devices that provide localized coupling. Such devices commonly have fixed construction, and the usage of PR-FZP enables to perform the focusing field control using a single unfocused ultrasonic transducer. This research implied the necessity to complete several tasks. Firstly, the design of PR-FZP was determined using Equations (1)–(3) and data of planned ultrasonic inspections presented in Table 1. As a result, the determined thickness of PR-FZP was 2.19 mm and the following radii of the acoustic lens were obtained: 5.87, 7.33, 8.98, 10.37, 11.60, and 12.71 mm. Secondly, the effectiveness of PR-FZP with the obtained design was verified via computer simulation using FEM modeling in COMSOL software. Results of computer modeling were evaluated via the determination of FWHM and FLHM values. Obtained result for FWHM (1.475 mm) demonstrated that the application of PR-FZP with a determined design has the ability to obtain the results with high lateral resolution. Thirdly, PR-FZP with a proper design was manufactured using 3D printing and PLA filament. Dimensions of the manufactured acoustic lens were verified prior to its application in experimental verification. Finally, the manufactured PR-FZP with the proper design was verified via in-situ experiments. The set of testing blocks with FBH drilled at the depth of 20 mm was used in experiments. The application of PR-FZP showed significant improvement in ultrasonic imaging results relative to the cases when unfocused transducer is used. When PR-FZP was applied the relative errors of FBH diameters evaluation using the −6 dB drop method were not exceeded 4%, 3%, and 6% for FBH with diameters 5, 3, and 2 mm respectively. At the same time the SNR estimation for flaws was not lower than 25.7, 20.6, and 17.1 dB for FBH with diameters 5, 3, and 2 mm, respectively. Obtained experimental results confirm the efficiency of PR-FZP for robotic ultrasonic testing applications. The fact that excitation electronic signals with common parameters for ultrasonic testing were applied implies the possibility of PR-FZP application with the standard electronic units for ultrasonic non-destructive testing.

Obtained results can be used for the development of novel equipment which provides focused acoustic field generation for the needs of ultrasonic non-destructive testing. In the robotic ultrasonic inspections, the application of these acoustic lenses possesses an interest in devices that provide localized coupling. It is related to the fact that such lenses allow to perform flexible focused field control with the fixed thickness of coupling media. Also, these acoustic lenses application provides the acquisition of testing results with high lateral resolution and high signal-to-noise ratio. Also, PR-FZP have good manufacturability due to the fact that they can be produced using 3D printer. Furthermore, obtained results can serve as the basis for further research aimed at increasing the efficiency of PR-FZP application in ultrasonic non-destructive testing. One of the factors which affect such efficiency is the parameters of the applied excitation electronic signals. In this research the application of rectangular excitation signals is conditioned by the signal being one of the most common for the ultrasonic testing equipment. However, the form and duration of the applied type of ultrasonic signal can strongly affect the quality of testing results, especially in the case of PR-FZP application. Furthermore, the efficiency of ultrasonic imaging can be increased using the PR-FZP with advanced design (e.g., design of PR-FZP with multiple levels). Furthermore, PR-FZP can be improved by introducing reflecting layers on side faces of phase reversal regions of PR-FZP or matching layers on the interface between the lenses. However, all aforementioned changes in PR-FZP design cause the increase in the complexity of its manufacturing. Due to this, the development of advanced PR-FZP is reasonable only in the case of significant increase of the imaging efficiency using this lens. Further work is also to be related to the development of devices that provide localized coupling to allow for all possible advantages of PR-FZP application.

## Figures and Tables

**Table 1:** Parameters of planned ultrasonic inspection.

Parameter	Value
The central frequency of ultrasonic transducer	5.55 MHz
The diameter of ultrasonic transducer	25.4 mm
Thickness of coupling media	12 mm
Required depth of the focusing in testing specimen	20 mm
Speed of longitudinal waves in testing object	5900 m/s
Speed of longitudinal waves in coupling media	1500 m/s
Speed of longitudinal waves in PR-FZP material	2220 m/s

**Table 2:** Results of flaws size evaluation by using −6 dB drop method.

Block	A	B	C
Diameter of FBH in the block (mm)	5	3	2
Estimated size of the flaw for the cases without PR-FZP application (mm)	22.5	22	21.75
SNR for the cases without PR-FZP application (dB)	18.9	13.6	11.1
Estimated size of the flaw for the cases with PR-FZP application (mm)	5.13	3	1.87
SNR for the cases with PR-FZP application (dB)	26.6	20.7	17.6

**Table 3:** Results of flaws size evaluation by using −6 dB drop method.

Block	D				E				F			
FBH Diameter (mm)	5				3				2			
Defect number	1	2	3	4	1	2	3	4	1	2	3	4
FBH Size using PR-FZP (mm)	5.1	5.2	5.1	5	3	3.1	3.1	3	1.9	1.9	1.9	2
SNR using PR-FZP (dB)	25.7	25.9	26.6	26	20.7	21	20.6	21.1	17.1	17.1	17.5	17.3

## Data Availability

The data that support the findings of this study are available from the corresponding author upon reasonable request.
